# Adolescents’ ultra-processed food consumption status and its association with food literacy: a cross-sectional study in Chongqing, China

**DOI:** 10.3389/fnut.2025.1494896

**Published:** 2025-09-11

**Authors:** Jiaxin Guo, Ya Shi, Yu Su, Ke Jiang, Yaocheng Luo, Huiyi Zhang, Shengping Li, Zumin Shi, Liang Ran, Yong Zhao

**Affiliations:** ^1^College of Public Health, Chongqing Medical University, Chongqing, China; ^2^Research Center for Medicine and Social Development, Chongqing Medical University, Chongqing, China; ^3^Nutrition Innovation Platform-Sichuan and Chongqing, Chongqing, China; ^4^Research Center for Public Health Security, Chongqing Medical University, Chongqing, China; ^5^Disease Control and Prevention Center, Jiulongpo District People's Hospital, Chongqing, China; ^6^Department of Children Healthcare, Chongqing Health Center for Women and Children/Women and Children's Hospital of Chongqing Medical University, Chongqing, China; ^7^College of Health Sciences, Qatar University, Doha, Qatar; ^8^Health Management Center, First Affiliated Hospital of Chongqing Medical University, Chongqing, China; ^9^Chongqing Key Laboratory of Child Nutrition and Health, Children’s Hospital of Chongqing Medical University, Chongqing, China

**Keywords:** adolescents, Chongqing, food literacy, ultra-processed foods consumption, cross-sectional study

## Abstract

**Background:**

The consumption of ultra-processed foods (UPFs) is increasing globally and has become a prominent public health concern. We aimed to use a population-based study to examine the association between food literacy (FL) and its two subdomains with UPF consumption in adolescents.

**Methods:**

The online survey included 7,761 adolescents aged 11–17 from Chongqing, China. UPF consumption degree was assessed by the unhealthy eating subscale of the Healthy and Unhealthy Eating Behaviors Scale (HUEBS). FL was measured using the Food Nutrition Literacy in School-age Children (FNLQ-SC) questionnaire. FL and the two subdomains were categorized into quartiles, and linear regression was used to examine the association between them.

**Results:**

In fully adjusted regression models, the regression coefficients *β* (95% confidence interval) were 0.00, −0.68 (95% CI: −1.12, −0.24; *p* = 0.003), −0.69 (95% CI: −1.14, −0.24; *p* = 0.003), and −0.60 (95% CI: −1.06, −0.14; *p* = 0.012) across the FL quartiles. An inverse association between FL and UPF consumption score was observed only in girls, but not in boys. Among those with screen time ≥2 h/day, participants in the higher quartile of FL scores (Q3) exhibited lower scores in UPF consumption compared with those in quartile 1 (Q1) (*β* (95% CI) −1.35 (−2.00, −0.71), *p* < 0.05). There were significant interactions between FL quartiles and gender (*p* for interaction < 0.001) or screen time (*p* for interaction = 0.003) in relation to UPF consumption.

**Conclusion:**

This study suggests that high FL and the two subdomains were linked with a lower UPF consumption score in adolescents. Increasing FL among adolescents has the potential to enhance their decision-making on eating.

## Introduction

Investigating the level of food processing has become an increasing focus in recent years (1). The NOVA classification system is widely used to categorize foods based on the type and extent of industrial processing, with ultra-processed foods (UPFs) representing the highest level (2). UPFs are industrially manufactured products made from refined, low-cost ingredients, containing little to no whole foods, and often include additives such as stabilizers, artificial flavors, and coloring agents (3). They are highly palatable, energy-dense, and convenient, and now account for the majority of dietary energy intake in high and middle-income countries (4, 5). A recent study has shown that UPF sales increased across all countries between 2009 and 2019 (6). In the United States, data involving adolescents revealed a significant rise in the proportion of total energy intake from UPFs, increasing from 61.4% in 1999 to 67.0% in 2018 (7). Similarly, data from the China Nutrition and Health Survey indicated that per capita UPF consumption among Chinese adults quadrupled between 1997 and 2011 (8).

Meta-analysis stated that UPF consumption is an unhealthy eating behavior that is associated with multiple unhealthy outcomes (9). A Spanish birth cohort study of 2,377 mother–child pairs found that higher UPF intake during pregnancy was associated with intellectual impairment in early childhood (10). Cross-sectional evidence from Italy and South Korea suggests that higher UPF intake is associated with increased depressive symptoms, particularly among young adults and females (11, 12). Meanwhile, high UPF consumption is widely recognized as an unhealthy dietary behavior and has been linked to various adverse health outcomes in adolescents, including obesity (abdominal obesity/overweight), hypertension, sleep disorders, and dental caries (13–16). As adolescent health becomes a central focus of many national public health agendas, the marketing of unhealthy products—especially UPFs rich in fat, sugar, and salt—has emerged as a significant driver of health issues in this group (17).

Previous studies have revealed multiple factors that will affect UPF consumption in adolescents, including parental-related attitudes, behaviors, nutritional knowledge, and food literacy (18–21). Food literacy (FL) is defined as a collection of interrelated knowledge, skills, and behaviors required to plan, manage, select, prepare, and eat food to meet needs and determine intake (22), which can be considered a specific form of health literacy. FL focuses more on dietary practices compared with nutrition literacy (23). Moreover, FL comprises two key dimensions: (i) Cognition—the ability to acquire, comprehend, and internalize food- and nutrition-related knowledge; and (ii) Skills—the ability to make appropriate nutrition decisions and maintain a healthy diet (24). Existing studies have shown the correlation between people’s FL and dietary habits. A cross-sectional study in Japan has revealed that several aspects of FL are associated with the consumption of highly processed foods in adults (25). The “Eat Better Feel Better” program in Scotland improved caregiver FL and reduced processed food intake in families (26). Moreover, increased screen exposure and longer sedentary time have been positively associated with higher intake of UPFs (7, 27). Parental FL has been identified as a significant predictor of adolescents’ food choices in a multicountry study conducted across 10 Arab nations (19). Additionally, a cross-sectional survey involving 1,002 adolescents aged 11–19 years in Belgian secondary schools revealed that exposure to processed food marketing on social media was positively associated with adolescents’ self-reported consumption of sugar-sweetened beverages and other UPFs (28). Furthermore, higher levels of food literacy among adolescents have been consistently linked to healthier dietary patterns, including increased intake of nutritious foods (29). However, most studies have focused on the influence of parents’ and caregivers’ FL on children’s food choices and dietary behaviors. Given the complex and multifaceted effects of UPF consumption on public health, particularly among adolescents, it is crucial to explore the relationship between FL and UPF consumption in this population.

To fill the aforementioned research gap, this study aimed to: (i) examine the association between FL, including its two subdomains (cognition and skills), and UPF consumption using linear regression among adolescents aged 11–17 years in Chongqing, China; and (ii) explore potential interaction effects between FL and key demographic characteristics through subgroup analyses. We hypothesized that lower levels of FL and two subdomains (i.e., cognized and skills) are associated with higher levels of UPF consumption and that gender may moderate this association.

## Methods

### Study participants and procedures

This cross-sectional study was conducted between January 2023 and February 2023. The online survey platform “Questionnaire Star” was utilized, which is a professional online survey platform in China. We selected middle school students in 32 out of the 39 administrative areas in Chongqing as participants by randomization. The participants were required to meet several criteria: (1) aged 11–17 years, (2) students and their caregivers underwent an online informed consent process, and (3) be registered and/or reside in Chongqing. We excluded participants: (1) children with BMI-for-age Z-score (BAZs) beyond +6 or less than −4 were excluded (30). BMI was calculated from self-reported height and weight, (2) an unreasonably short time to complete the online questionnaire (<10 min), and (3) reported “do not know of their main caregiver’s education.” Eventually, 7,761 participants were included in this analysis.

This study was conducted with the assistance of the local Municipal Education Commission, which distributed the questionnaire link to each regional school health worker. The school health worker then passes the online questionnaire on to the class group. Before accessing the questionnaire, participants were presented with a detailed description of the study’s purpose and the voluntary nature of participation. Informed consent was obtained electronically, and only after providing consent from students and their caregivers were students directed to complete the questionnaire anonymously, which included demographic details, such as age, gender, and single-child status, FL assessment, and dietary behaviors. The caregiver status was verified by requiring the relationship confirmation, and assistance was provided when needed to guide students in filling out the questionnaire. The study was approved by the Ethics Committee of Chongqing Medical University (Approval Number: 2021041).

### Food literacy assessment

FL among adolescents was assessed using the Food Nutrition Literacy in School-age Children (FNLQ-SC) questionnaire (24), which has been validated and proven to be reliable in Chinese children, with a Cronbach’s alpha of 0.851 and a Kaiser–Meyer–Olkin (KMO) value of 0.929 in this study. The questionnaire comprises 43 items, 15 of which focus on cognition (nutrition knowledge), such as “I will get information related to nutrition and health actively,” and 28 items on skills (food choice, cooking, and food-related skills), “I select food from a health perspective.” It is a self-report tool that uses a 5-point Likert scale (“disagree” to “strongly agree”) to evaluate, and single-choice questions are scored based on correctness (1 point) or errors (0 points). Total score ranges from 0 to 145, with higher scores indicating higher FL levels among students. Specifically, the cognition subscale has a score range of 0–51, while the skills subscale ranges from 0 to 94. Subsequently, the total FL score and its two subdomains were divided into quartiles (Q1, Q2, Q3, and Q4) for further analysis and comparison.

### Ultra-processed food consumption extent assessment

The Healthy and Unhealthy Eating Behaviors Scale (HUEBS) (31) was a valid tool to assess the extent to which the participants generally consume healthy and unhealthy food items. However, the HUEBS is based on Canada’s Food Guide. Due to the difference in food culture between the East and the West, this scale may not be suitable for the Chinese. Thus, the HUEBS was localized based on the Dietary Guidelines for Chinese Residents (32) to assess the extent of healthy and unhealthy food consumption among the Chinese. Examples of healthy food items included: Examples of healthy food items included: “whole grains (e.g., brown rice, buckwheat, quinoa, oats),” “fruits,” “vegetables,” and examples of food items that should be consumed in moderation included: “frozen sweet products (e.g., ice cream, popsicle),” “packaged preserved fruits,” “chocolate, and/or candy.” The values for KMO (0.902) and Barlett’s test of sphericity (*p* < 0.001) indicated that the scale was adequate for conducting Principal Component Analysis. All items of the subscale demonstrated rotation factor loadings greater than 0.40, with the variables primarily reflecting the characteristics of their respective factors. Cronbach’s alpha also revealed that the subscales had good internal consistency (*α* = 0.816 for healthy eating and *α* = 0.831 for unhealthy eating).

The study used the unhealthy eating subscale of HUEBS to assess the degree of UPF consumption. Two trained dietitians categorized the food products, with a third dietitian assisting in cases of mismatch to ensure the items of the subscale are classified as UPFs according to the NOVA classification system (2), the highest degree of processed (group 4). Participants were asked to indicate the extent to which they generally consume each food using a scale ranging from 1 (Never) to 7 (≥3 times/day). Composite scores for UPF consumption were created by summing the respective items of the subscale. A score was calculated by the sum of answers, varying from 7 to 49 points (33). The higher score reflected a higher UPF consumption degree and a greater unhealthy eating behavior (31).

### Adolescent demographic and lifestyle survey

Participants’ demographic information was collected through a structured questionnaire, including age, gender, ethnicity (Han and minority), residence (urban and rural, classified by the National Bureau of Statistics) (34), only child (yes or no), boarding schools, and caregiver education (low, elementary school, and below; medium, junior high school; and high, high school, or above). The self-reported weight and height were converted to BAZs using the World Health Organization (WHO) AnthroPlus software, and the classification standard is under the WHO standard (35): <−2 represents underweight, ≥ − 2 to 1 represents normal, ≥1 to 2 represents overweight, and >2 represents obesity. The video screen time survey primarily collected self-reported data on the amount of time participants spent watching TV, playing games, and browsing on mobile phones daily, categorized as healthy (<2 h/day) or unhealthy (≥2 h/day) (27). Sedentary time was self-reported by the Physical Activity Questionnaire (36), which was assessed by the question “How much time do you usually spend sitting on a typical day?” The date of sedentary behavior was classified as <2 h/day and ≥2 h/day (37).

### Statistical analysis

Frequency and proportion (%) were used to describe categorical variables, and mean and standard deviation (SD) were used to describe continuous variables. Participants’ demographic characteristics were presented according to quartiles of FL score and compared using a chi-squared test for categorical variables (e.g., gender, ethnicity, residence, etc.) or analysis of variance (ANOVA) for continuous variables (age). With UPF scores as the primary outcome variable, linear regression analysis was conducted to estimate the relationships among quartiles of FL (including its subdomains). Since weight status, screen time, and sedentary time are associated with adolescents’ UPF consumption (38, 39), they were controlled in the linear regression analysis to eliminate these effects. Significant variables in the single-factor analysis were also controlled as covariates. Multicollinearity among covariates in the multivariable-adjusted model was assessed using variance inflation factors (40). All variance inflation factors were below 5, indicating low collinearity. Each group had the following three models established: model 1 was not adjusted; model 2 was adjusted for age, gender, BAZs, boarding school, residence, and caregiver education; and model 3 was further adjusted for sedentary time and screen time. In the stratified analysis, we examine the interaction effect by introducing interaction terms into the regression model. All analyses were performed using STATA version 17.1 (STATA Corporation, College Station, TX, USA); *p*-value < 0.05 was considered statistically significant.

## Results

### Demographics of the study sample

Table 1 presents the characteristics of the participant sample categorized by FL quartiles. The study included a total of 7,761 individuals, comprising 3,716 boys (47.9%) and 4,045 girls (52.1%), with a mean age of 14.00 ± 0.98 years. 74.0% of the students fell within the normal weight range. The majority of the participants were of Han ethnicity (95.5%). 35.1% were boarding students, 58.9% resided in urban areas, and 23.8% were only children. Most participants’ primary caregivers had attained a secondary education level (45.6%). Additionally, 20.4% reported daily sedentary time exceeding 2 h, while 45.4% reported daily screen time exceeding 2 h.

**Table 1:** Demographic characteristics of participants based on quartiles of food literacy scores (*N* = 7,716).

Variables*	Total	Q1	Q2	Q3	Q4	*p*-value
	*N* = 7,761	*N* = 2,035	*N* = 2,007	*N* = 1,829	*N* = 1,890	
Age	14.00 (0.98)	14.01 (1.00)	14.05 (0.98)	13.98 (0.97)	13.94 (0.96)	0.005
Adolescents’ weight status						0.180
Normal	5,747 (74.0%)	1,472 (72.3%)	1,493 (74.4%)	1,375 (75.2%)	1,407 (74.4%)	
Underweight	263 (3.4%)	86 (4.2%)	60 (3.0%)	56 (3.1%)	61 (3.2%)	
Overweight	1,042 (13.4%)	269 (13.2%)	275 (13.7%)	251 (13.7%)	247 (13.1%)	
Obese	709 (9.1%)	208 (10.2%)	179 (8.9%)	147 (8.0%)	175 (9.3%)	
Gender						<0.001
Boy	3,716 (47.9%)	1,090 (53.6%)	943 (47.0%)	817 (44.7%)	866 (45.8%)	
Girl	4,045 (52.1%)	945 (46.4%)	1,064 (53.0%)	1,012 (55.3%)	1,024 (54.2%)	
Ethnicity						0.210
Han	7,415 (95.5%)	1,934 (95.0%)	1,912 (95.3%)	1,748 (95.6%)	1,821 (96.3%)	
Others	346 (4.5%)	101 (5.0%)	95 (4.7%)	81 (4.4%)	69 (3.7%)	
Boarding school						0.002
Yes	2,723 (35.1%)	779 (38.3%)	701 (34.9%)	627 (34.3%)	616 (32.6%)	
No	5,038 (64.9%)	1,256 (61.7%)	1,306 (65.1%)	1,202 (65.7%)	1,274 (67.4%)	
Residence						<0.001
Urban	4,570 (58.9%)	1,136 (55.8%)	1,171 (58.3%)	1,089 (59.5%)	1,174 (62.1%)	
Rural	3,191 (41.1%)	899 (44.2%)	836 (41.7%)	740 (40.5%)	716 (37.9%)	
Only child						0.620
Yes	1,847 (23.8%)	491 (24.1%)	459 (22.9%)	432 (23.6%)	465 (24.6%)	
No	5,914 (76.2%)	1,544 (75.9%)	1,548 (77.1%)	1,397 (76.4%)	1,425 (75.4%)	
Caregiver education						<0.001
Low	2,070 (26.7%)	652 (32.0%)	581 (28.9%)	414 (22.6%)	423 (22.4%)	
Middle	3,539 (45.6%)	880 (43.2%)	904 (45.0%)	889 (48.6%)	866 (45.8%)	
High	2,152 (27.7%)	503 (24.7%)	522 (26.0%)	526 (28.8%)	601 (31.8%)	
Sedentary time						0.130
<2 h	6,178 (79.6%)	1,602 (78.7%)	1,585 (79.0%)	1,451 (79.3%)	1,540 (81.5%)	
≥2 h	1,583 (20.4%)	433 (21.3%)	422 (21.0%)	378 (20.7%)	350 (18.5%)	
Screen time						<0.001
<2 h	4,235 (54.6%)	819 (40.2%)	1,039 (51.8%)	1,077 (58.9%)	1,300 (68.8%)	
≥2 h	3,526 (45.4%)	1,216 (59.8%)	968 (48.2%)	752 (41.1%)	590 (31.2%)	

*Data are presented as mean (SD) for continuous measures, and *n* (%) for categorical measures.

### FL quartiles and their association with demographic characteristics

Across the different FL quartiles, FL scores were 82.6 ± 7.8, 94.6 ± 2.3, 102.6 ± 2.6, and 116.6 ± 6.9, respectively, Q1 to Q4. As shown in Table 1, the distributions of age, gender, boarding school, residence, caregiver education, and children’s screen time were significantly different among the four FL score groups (quartiles, Q1–Q4) (*p* value < 0.05). In the Q1 group of the FL score, participants were slightly older. The proportion of boys was significantly higher in Q1, while girls predominated in Q2–Q4. From Q1 to Q4, there was an increasing proportion of non-boarding students and adolescents residing in urban areas, along with a higher proportion of caregivers with a higher education level. Conversely, the proportion of individuals reporting daily screen time of ≥2 h or more decreased.

### Adolescents’ UPF consumption degree

**Figure 1:**
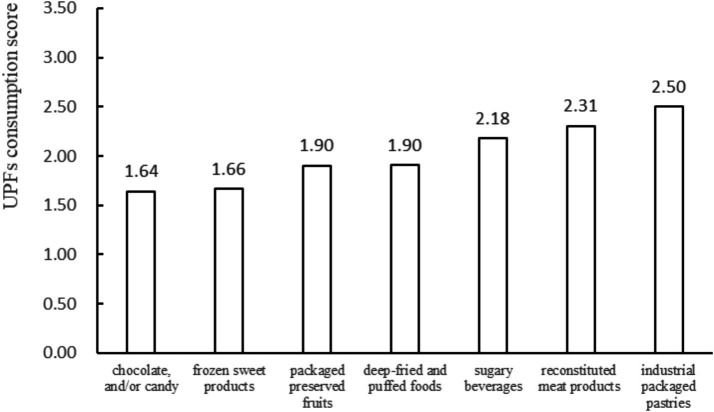
Overview of various ultra-processed foods scores among adolescents.

Figure 1 displays the scores for different categories of UPF. The scores, ordered from lowest to highest, were: chocolate and/or candy (1.64 ± 1.23), frozen sweet products (1.66 ± 1.23), packaged preserved fruits (1.90 ± 1.50), deep-fried and puffed foods (1.90 ± 1.34), sugary beverages (2.18 ± 1.49), reconstituted meat products (2.31 ± 1.54), and industrial packaged pastries (2.50 ± 1.55). Among them, industrial packaged pastries, reconstituted meat products, and sugary beverages are the three UPF groups with the highest consumption score.

### Association between FL and UPF consumption degree

Adolescents’ FL levels were negatively associated with UPF consumption scores (Table 2). After adjusting for sociodemographic factors (model 1), the *β* coefficients (95% confidence intervals) were 0, −0.80 (95% CI: −1.25, −0.37, *p* < 0.001), −0.90 (95% CI: −1.35, −0.45, *p* < 0.001), and −0.91 (95% CI: −1.36, −0.46, *p* < 0.001) across the FL quartiles. However, when further adjusting for lifestyle factors (model 3), the *β* coefficients decreased by 15.00, 23.33, and 34.06%, respectively, and the associations were attenuated. When analyzing the two FL subdomains, model 2 showed that participants in the highest quartiles (Q4) of cognitive scores had lower UPF consumption scores compared to those in the first quartile (Q1), with β coefficients (95% CI) of −0.59 (95% CI: −1.03 -0.14, *p* = 0.011). After further adjustment for lifestyle factors, the association between the cognitive dimension of FL and UPFs weakened. For the skills domain, participants’ scores were negatively associated with UPF consumption scores in model 2. The effect estimates for the second to fourth quartiles (Q2–Q4) still decreased (by 14.89, 22.55, and 44.43%, respectively) in model 3.

**Table 2:** Association between quartiles of food literacy categories and ultra-processed food consumption score.

Quartiles of food literacy score	Model 1^a^		Model2^b^		Model 3^c^	
	*β* coefficients^d^	*p-*value	*β* coefficients	*p-*value	*β* coefficients	*p*-value
	(95% CI)		(95% CI)		(95% CI)	
Food Literacy						
Q1	0.00		0.00	0.00	0.00	
Q2	−0.82 (−1.26, −0.38)	<0.001	−0.80 (−1.25, −0.37)	<0.001	−0.68 (−1.12, −0.24)	0.003
Q3	−0.93 (−1.38, −0.49)	<0.001	−0.90 (−1.35, −0.45)	<0.001	−0.69 (−1.14, −0.24)	0.003
Q4	−0.98 (−1.42, −0.54)	<0.001	−0.91 (−1.36, −0.46)	<0.001	−0.60 (−1.06, −0.14)	0.012
Cognized						
Q1	0.00	0.00	0.00	0.00	0.00	
Q2	−0.56 (−0.99, −0.13)	0.011	−0.51 (−0.95, 0.08)	0.020	−0.45 (−0.88, −0.02)	0.040
Q3	−0.92 (−1.35, −0.48)	<0.001	−0.85 (−1.29, −0.41)	<0.001	−0.75 (−1.19, −0.31)	0.001
Q4	−0.70 (−1.14, −0.26)	0.002	−0.59 (−1.03, −0.14)	0.011	−0.37 (−0.82, 0.08)	0.109
Skill						
Q1	0.00	0.00	0.00	0.00	0.00	
Q2	−0.96 (−1.38, −0.53)	<0.001	−0.94 (−1.37, −0.52)	<0.001	−0.80 (−1.23, −0.37)	<0.001
Q3	−1.04 (−1.47, −0.60)	<0.001	−1.02 (−1.45, −0.58)	<0.001	−0.79 (−1.23, −0.35)	<0.001
Q4	−0.76 (−1.21, −0.31)	0.001	−0.72 (−1.18, −0.26)	0.002	−0.40 (−0.86, 0.07)	0.102

a Model 1 unadjusted.

b Model 2 adjusted for age, BAZs, gender, boarding school, residence, and caregiver education.

c Model 3 further adjusted for sedentary time and screen time.

d *β* coefficients and 95% CI were derived from linear regression.

### Stratified analysis

The stratified analysis results show that higher FL levels are generally associated with a decrease in UPF consumption levels; however, significant demographic differences were observed (Table 3). Significant interactions were detected between FL and gender (*p* for interaction<0.001) and screen time (*p* for interaction = 0.003). An inverse association between FL and UPF consumption was observed in girls but not in boys. Furthermore, when screen time ≥2 h/day, we observed a negative correlation between FL and UPF consumption scores. Participants in high quartile FL scores (Q3) exhibited lower scores in UPF consumption compared with those in Q1 (*β* (95% CI) -1.35 (−2.00, −0.71), *p* < 0.05). No significant interactions were observed for boarding school status, residential location, caregiver education level, or sedentary time.

**Table 3:** Stratified analysis of the association between quartiles of food literacy and ultra-processed food consumption.

Variables	Quartiles of FL				*p* for trend	*p* for interaction
	Q1	Q2	Q3	Q4		
Gender						
Boy	0.00	−0.25 (−0.85, 0.35)	−0.55 (−1.18, 0.07)	−0.10 (−0.72, 0.51)	0.495	<0.001
Girl	0.00	−1.50 (−2.11, −0.89)	−1.45 (−2.07, −0.84)	−1.89 (−2.51, −1.18)	<0.001	
Boarding school						
Yes	0.00	−0.89 (−1.65, −0.14)	−0.83 (−1.60, −0.06)	−0.69 (−1.47, −0.09)	0.085	0.771
No	0.00	−0.78 (−1.30, −0.26)	−0.98 (−1.51, −0.45)	−1.07 (−1.59, −0.55)	0.001	
Residence						
Urban	0.00	−0.89 (−1.44, −0.34)	−0.90 (−1.46, −0.33)	−1.34 (−1.90, −0.79)	<0.001	0.090
Rural	0.00	−0.75 (−1.43, −0.08)	−1.02 (−1.72, −0.32)	−0.40 (−1.10, 0.30)	0.147	
Caregiver education						
Low	0.00	−0.77 (−1.59, 0.05)	−0.67 (−1.57, 0.24)	−0.62 (−1.52, 0.27)	0.1169	0.755
Middle	0.00	−1.05 (−1.70, −0.40)	−1.21 (−1.86, −0.56)	−0.99 (−1.65, −0.33)	0.003	
High	0.00	−0.47 (−1.26, 0.31)	−0.62 (−1.40, 0.17)	−1.03 (−1.79, −0.27)	0.008	
Sedentary time						
<2 h	0.00	−0.77 (−1.25, −0.29)	−0.93 (−1.43, −0.44)	−0.82 (−1.31, −0.34)	0.001	0.483
≥2 h	0.00	−1.05 (−1.99, −0.11)	−0.97 (−1.94, 0.02)	−1.61 (−2.60, −0.62)	0.003	
Screen time						
<2 h/day	0.00	−0.83 (−1.45, −0.21)	−0.27 (−0.88, 0.35)	−0.79 (−1.38, −0.19)	0.076	0.003
≥2 h/day	0.00	−0.52 (−1.12, 0.08)	−1.35 (−2.00, −0.71)	−0.26 (−0.96, 0.43)	0.033	

*Model adjusted for age, BAZs, gender, boarding school, residence, and caregiver education.

## Discussion

We conducted a cross-sectional study in Chongqing, China, examining the association between UPF consumption score and FL level, including cognition and skills, among junior high school students. Consistent with our hypothesis, FL and its two subdomains were negatively correlated with the UPF consumption score. Moreover, the FL level had significant interactions with gender and screen time. The negative association between FL and UPF score was only observed among girls. When exposed to 2 h or more of screen time, we observed that participants in the high quartile FL scores (Q3) exhibited reduced scores in UPF consumption compared with those in Q1.

In terms of UPF consumption, we found that industrial packaged pastries, reconstituted meat products, and sugary beverages are the most commonly consumed UPF groups among middle school students, which is consistent with previous studies. One study showed that among American working adults, the primary UPF consumption was from desserts and sweets (20.8%), followed by chips, crackers, and other related products (16.5%) (41). Another study involving Chinese primary and secondary school students (*N* = 1,274) has indicated that the three most prevalent UPFs were pastries (80.5%), confectionery (64.6%), and fried puffed food (53.9%) (42). A national study in Belgium found that the products contributing most to UPF consumption were processed meat (14.3%), pastries (8.9%), and soft drinks (6.7%) (43). Middle school students are at a pivotal stage of adolescent development, and UPF consumption has the potential to impede their physiological maturation and overall growth trajectory. Hence, it is imperative to mitigate or rectify poor dietary habits, cultivate healthy eating practices, and incorporate vegetables, fruits, soy products, and dairy items, among others, as supplementary sources of nutrition in addition to regular meals.

FL is considered important in shaping dietary habits. A previous study of adults reported a positive association exists between nutrition knowledge and consumption of vegetables, fruits, cereals, or fish in some core food groups, which is more consistent with public health guidelines, but inversely correlated with consumption of sweetened drinks and fat (44). Several other studies also reported a negative correlation in adults between food skills (food preparation skills and behaviors, cooking skills) and highly processed convenience food items (e.g., ready meals) (45, 46). However, there is limited evidence regarding the association between FL and UPF consumption among adolescents. In our study, we found that adolescents’ FL, including both cognition and skill subdomains, was negatively correlated with their UPF consumption score. Adolescents with elevated FL exhibited low UPF scores. Nutrition education can improve knowledge and, in turn, positively influence dietary intake (47, 48). Furthermore, adolescents with advanced culinary skills are markedly inclined to engage in cooking activities enthusiastically and experience a sense of pride in their achievements (49). A previous study also highlighted that individuals with high skills in food selection and preparation are more inclined to choose natural foods (e.g., raw meat, poultry, and vegetables) over UPFs (50). Therefore, possessing basic nutrition knowledge and culinary skills is essential for guiding adolescents in making healthier food choices.

In our research, age and gender are important factors influencing adolescents’ FL levels. The mean age of the quartile 1 (Q1) group for FL is slightly higher than that of the quartile 4 (Q4) group in this study. This difference may be attributed to increased academic stress and greater autonomy in food choices as adolescents age, which could lead to less healthy dietary behaviors, such as consumption of sugar-sweetened beverages (21). In contrast, younger children may benefit from more structured dietary environments at home or school, which could contribute to higher FL scores (51). Therefore, further research is needed to clarify the role of age in the relationship between FL and the UPF consumption. In addition, we found that the distribution of FL quartiles differs markedly between genders. A higher proportion of girls have higher FL quartile scores, showing girls had higher means of FL ratings compared to boys in this study. This may be related to female body image concerns and body weight idealism objectives, particularly in adolescence (52). These factors may lead to greater involvement and preoccupation with the energy and nutrition content of the food they eat among girls (53). Meanwhile, the negative association between FL level and UPF consumption score was observed only in girls but not in boys. This finding could be attributed to girls having more attention to food knowledge than boys (29). One study has shown that compared with boys, girls are more involved in food-related tasks, such as reading food labels more frequently, and tend to avoid choosing unhealthy foods (54). Moreover, boys may receive higher calorie meals than girls, typically from unhealthy sources, potentially increasing their preference for unhealthy foods (55). Future studies should carefully consider these gender differences in knowledge and food skills and elucidate the mechanisms of the gender difference in the association between UPFs and FL.

Screen time (27) refers to the time spent watching TV, playing games, and browsing on mobile phones per day. We found that screen time significantly modified the association between FL and UPF consumption. When exposed to 2 h or more of screen time, participants with higher quartiles of FL scores (Q3) significantly reduced UPF consumption score. Although the reason for this is unclear, there may be a possibility related to nutrition education and social support. For example, in Australia, incorporating nutrition education into the game improved children’s overall nutrition knowledge, which may help them provide positive feedback about their diet (56). Additionally, a series of healthy dietary and physical activity interventions have been successfully integrated into intelligent devices to improve diet and physical activity behaviors (57, 58). However, we could not examine the potential impact of the screen environment because the information was not available in this study. Therefore, further research would be needed to clarify this aspect of the association between screen time and UPF consumption, and to adopt a stratified improvement strategy to customize the FL intervention for adolescents.

The association between UPF consumption and a range of health issues is well-established (13–16). Despite the Chinese government’s proactive measures, such as the Chinese Dietary Guidelines (CDG), UPF consumption of these foods continues to escalate in China. Evidently, improving FL among adolescents has the potential to enhance their decision-making on food choices, understanding of food labeling, and adoption of healthy cooking practices (21, 25). Thus, these findings hold significant implications for policymakers, researchers, and other stakeholders in society. Several strengths in our study should be highlighted. One of these is the focus on a substantial sample of adolescents, with nearly equal proportions of males and females from diverse regions across Chongqing, China. Additionally, we conducted a comprehensive assessment of their FL, including nutrition knowledge and skills (preparing, cooking, and intake), and utilized a scale (HUEBS) modified according to the Chinese Nutrition Society (32) to assess the extent of UPF consumption. Furthermore, two trained dietitians categorized the food products for NOVA classification, with a third dietitian assisting in cases of discrepancy. This study also holds significant policy implications.

Nevertheless, some limitations warrant mention. First, online and self-reported surveys inherently introduce information biases. However, stringent quality control measures were implemented throughout the process, and the participant samples were strictly screened. Second, cross-sectional survey data did not permit a reliable inference of causality. Longitudinal studies are necessary to further examine the association between FL and UPF consumption. Third, our inability to access comprehensive information, compounded by participants’ reluctance to disclose sensitive details, such as household economic status (59), eating location (60), and patterns of neighborhood food outlets (61), limited our capacity to investigate potential internal and external factors linked to UPF consumption. Future research should incorporate a broader range of factors, including strategies for overcoming such reluctance, to enable a more nuanced and thorough analysis.

## Conclusion

The results of this cross-sectional study suggest that industrial packaged pastries, reconstituted meat products, and sugary beverages are the most commonly consumed UPF groups by middle school students. FL and its two subdomains were found to be negatively associated with UPF consumption scores among adolescents. Additionally, we observed significant interactions between FL levels, gender, and screen time. These findings highlight the potential of improving FL to enhance adolescents’ food-related decision-making, which has important implications for policymakers, researchers, and other relevant stakeholders. However, given the influence of various behavioral and sociodemographic factors (e.g., caregiver feeding practices, family behavior, and eating environments), further longitudinal studies are necessary to clarify the long-term relationship between FL and UPF consumption.

## Data Availability

The datasets generated and/or analyzed during the current study are not publicly available due to funding requirements but are available from the corresponding author on reasonable request.
